# Genetic and Nongenetic Risk Factors for Breast Cancer Risk Estimation

**DOI:** 10.1001/jamanetworkopen.2025.5804

**Published:** 2025-04-18

**Authors:** Wenji Guo, James L. Li, Julian McClellan, Atara Ntekim, Oladosu Ojengbede, Temidayo Ogundiran, Abayomi Odetunde, John Obafunwa, Abiodun Popoola, Paul Ndom, Antony Gakwaya, Nilanjan Chatterjee, Elisabeth Sveen, Toshio F. Yoshimatsu, Yonglan Zheng, Olufunmilayo I. Olopade, Dezheng Huo

**Affiliations:** 1 Section of Hematology and Oncology, Department of Medicine, The University of Chicago, Chicago, Illinois; 2 Department of Public Health Sciences, The University of Chicago, Chicago, Illinois; 3 Department of Radiation Oncology, College of Medicine, University of Ibadan, Ibadan, Nigeria; 4 Center for Population and Reproductive Health, College of Medicine, University of Ibadan, Ibadan, Nigeria; 5 Department of Surgery, College of Medicine, University of Ibadan, Ibadan, Nigeria; 6 Institute for Advanced Medical Research and Training, College of Medicine, University of Ibadan, Ibadan, Nigeria; 7 Department of Pathology and Forensic Medicine, Lagos State University Teaching Hospital, Lagos, Nigeria; 8 Department of Radiation and Clinical Oncology, Lagos State University Teaching Hospital, Lagos, Nigeria; 9 Yaounde General Hospital, Yaounde, Cameroon; 10 Department of Surgery, King Ceasor University, Kampala, Uganda; 11 Department of Biostatistics, Johns Hopkins University, Baltimore, Maryland; 12 Center for Global Health, The University of Chicago, Chicago, Illinois

## Abstract

**Importance:**

Most breast cancers in Africa are diagnosed at advanced stages. Improved risk prediction tools to optimize screening and earlier diagnosis are urgently needed.

**Objective:**

To build a comprehensive breast cancer risk estimation model by integrating a polygenic risk score (PRS), pathogenic variants (PVs) in high- or moderate-penetrance genes, and a questionnaire-based risk calculator.

**Design, Setting, and Participants:**

This multicenter case-control study initially enrolled women in Nigeria in 1998 and expanded to Cameroon and Uganda in 2011; enrollment ended in 2018. Women with breast cancer (hereafter cases) were enrolled through hospital oncology units, whereas women without breast cancer (hereafter controls) were recruited from other outpatient clinics and the community. Participants whose genetic data were used in PRS development were excluded from the development of the comprehensive breast cancer risk estimation model. Analyses were performed from September 2023 to January 2025.

**Exposures:**

Lifetime absolute risk estimation models that integrated a PRS only (previously developed using data from women of African ancestry and European ancestry), PRS plus PVs in high- or moderate-penetrance genes (*BRCA1*, *BRCA2*, *PALB2*, *ATM*, *CHEK2*, *TP53*, *BARD1*, *RAD51C*, and *RAD51D*), epidemiologic risk factors only (ascertained from NBCS questionnaires), and a combined model containing these 3 components.

**Main Outcomes and Measures:**

Lifetime absolute risk of breast cancer was estimated, accounting for an association between family history and genetic factors. Participants’ lifetime estimated absolute risk was categorized by the following risk thresholds: lower than 3%, 3%, 5%, and 10% or higher.

**Results:**

A total of 1686 women, of whom 996 were cases (mean [SD] age at enrollment, 49.5 [12.2] years) and 690 were controls (mean [SD] age at enrollment, 41.5 [13.8] years), were included in the main analyses. The age-adjusted area under the receiver operating characteristic curve (AUROC) was 0.579 (95% CI, 0.549-0.610) for the PRS only model and 0.609 (95% CI, 0.579-0.638) for the PRS plus PV model. In the combined model containing both genetic and nongenetic risk factors, age-adjusted AUROC increased to 0.723 (95% CI, 0.698-0.748). Using a threshold of 10% or higher lifetime absolute risk, the combined model classified 12.0% of cases (120) as high risk compared with 3.7% of cases (37) using the epidemiologic factors only model and 5.0% of cases (50) using the PRS plus PV model.

**Conclusions and Relevance:**

In this case-control study, a breast cancer risk estimation model was developed that combines genetic and nongenetic factors and refines a previous model that includes epidemiologic risk factors. Further development and validation of this model are necessary to advance breast cancer risk assessment in sub-Saharan Africa.

## Key Points

**Question:**

Can adding genetic information to risk estimation models based on epidemiologic factors improve breast cancer risk assessment for women in sub-Saharan Africa?

**Findings:**

In this case-control study of 1686 women in Nigeria, Cameroon, and Uganda, a breast cancer risk estimation model was developed that combines single-nucleotide variants and carrier status of high or moderate penetrance pathogenic variants with epidemiologic risk factors. This combined model achieved higher accuracy.

**Meaning:**

The comprehensive breast cancer risk estimation model developed in this study has the potential to advance breast cancer control efforts by improving risk-stratified targeted screening and early diagnosis in sub-Saharan Africa.

## Teaser

This case-control study describes the development of a breast cancer risk estimation model that incorporates a polygenic risk score, pathogenic variant carrier status, and epidemiologic risk factors to estimate the risk of breast cancer among women in sub-Saharan Africa.

## Introduction

Breast cancer is the most common cancer in women worldwide.^1^ There is no population-based breast cancer screening program in any African country, partly due to the young age structure of the populations and lower incidence rates. However, due to the prevalence of advanced-stage disease at diagnosis, the mortality to incidence ratio is high, with mortality rates comparable to those in high-income countries.^2^ To prevent premature deaths from breast cancer diagnosed at advanced stages, early diagnosis and prompt access to effective therapies are paramount. Personalized risk assessment, in combination with risk-stratified screening for early diagnosis and biomarker-informed treatment, has the potential to accelerate progress and reduce breast cancer mortality in Nigeria and similar low- to middle-income countries.^3^ However, more rigorous research is needed before implementing clinical guidelines that were not derived using data from indigenous African populations.

Multiple factors are associated with breast cancer susceptibility, including lifestyle, reproductive, and hormonal factors as well as both rarer pathogenic variants (PVs) in high- or moderate-penetrance genes and more common single-nucleotide variants (SNVs). Comprehensive breast cancer risk estimation models, such as BOADICEA (Breast and Ovarian Analysis of Disease Incidence and Carrier Estimation Algorithm), have demonstrated improved breast cancer risk stratification when all of these factors are jointly considered.^4^ However, the majority of these existing models have been developed in populations of European ancestry^4,5,6^ and have limited generalizability to populations of African ancestry due to differences in the underlying lifestyle or reproductive risk factor distributions,^7^ linkage disequilibrium patterns, allele frequencies, and breast cancer incidence rates. Furthermore, high carrier frequencies of PVs in high- or moderate-penetrance genes have been reported in patients with breast cancer who were unselected for family history and age in Nigeria, Cameroon, Uganda, and Ghana.^8,9,10^ There is a paucity of data from populations of African ancestry in genome-wide association study datasets. Polygenic risk scores (PRSs) that were developed in populations of European ancestry have a more modest predictive value when applied to populations of African ancestry due to differences in linkage disequilibrium patterns and allele frequencies across these populations.^11,12^ To optimize breast cancer risk stratification for women in sub-Saharan Africa, it is crucial that the risk estimation models are based on data collected from African countries. Nigeria is the largest country in Africa, with an age-specific incidence rate of breast cancer estimated to be as high as 64.6 of 100 000 people and rising.^13^

Several questions remain unanswered regarding the clinical utility of currently available tools for breast cancer risk assessment in indigenous African women, such as whether adding genetic information to risk estimation models based on epidemiologic risk factors can improve risk assessment. In this case-control study, we aimed to build a comprehensive breast cancer risk estimation model by integrating a PRS developed for women of African ancestry, PVs in high- or moderate-penetrance genes, and a Nigerian Breast Cancer Study (NBCS) questionnaire–based risk calculator.^7^

## Methods

This study received approval from the institutional review board of each study site in Nigeria, Uganda, and Cameroon. All participants provided written informed consent. We followed the Strengthening the Reporting of Observational Studies in Epidemiology (STROBE) reporting guideline.

### Study Population

All study participants were women aged 18 years or older. The NBCS started enrolling women with breast cancer (hereafter cases) and women without breast cancer (hereafter controls) in Nigeria in 1998 and, in 2011, enrollment was extended to Cameroon and Uganda using the same questionnaire and protocol. The study design is described in detail elsewhere.^9,14,15^ Briefly, cases were identified through University College Hospital Ibadan, a referral center for other hospitals in Nigeria. Cases were recruited through the surgical oncology and radiotherapy units of University College Hospital. Controls were enrolled through general outpatient and ophthalmology clinics as well as from several communities randomly selected from a list of all communities in the catchment area of University College Hospital. Starting in 2014, cases were enrolled at Lagos State University Teaching Hospital using the same NBCS questionnaire.^16^

In Uganda, cases were recruited from the breast and endocrine units of the Department of Surgery of Mulago Hospital in Makerere University in Kampala, a national referral hospital. Controls were recruited randomly from the general outpatient clinics and inpatient surgical services of Mulago Hospital. In Cameroon, cases were enrolled from the Department of Medical Oncology of Yaoundé General Hospital, and controls were randomly recruited from general medicine and obstetrics and gynecology clinics.

### Nongenetic and Genetic Risk Factors

Trained interviewers administered structured questionnaires, measured height and weight, and obtained blood samples. The questionnaires covered demographics, lifestyle, family history of breast cancer, history of benign breast disease, and menstrual or reproductive history. Based on findings from the development of the initial NBCS breast cancer risk estimation model,^7^ the following breast cancer risk factors were included in the present model: age at menarche, parity, total months of breastfeeding, history of benign breast disease, family history of breast cancer in first-degree relatives, height, body mass index (BMI; calculated as weight in kilograms divided by height in meters squared), and alcohol intake. Alcohol consumption was defined as having ever consumed alcoholic beverages at least once a week for 6 continuous months or longer. Status of benign breast disease was assessed by the question, “Has a doctor ever told you that you had benign breast disease, such as a noncancerous cyst or a breast lump?”

Genotype data for women of African ancestry were obtained from The Root (GWAS [Genome-Wide Association Study] of Breast Cancer in the African Diaspora) Consortium, Breast Cancer Association Consortium, and African-Ancestry Breast Cancer Genetic Consortium. The samples were genotyped using different arrays (including HumanOmni 2.5-8v1 array, OncoArray, and Multi-Ethnic Genotyping Array; Illumina Inc). The genotyping and quality control procedures have been described previously.^17,18,19^ The BROCA cancer risk panel and the 30-gene hereditary cancer risk panel (Color Genomics) were used for detection of pathogenic and likely PVs. Sequencing was performed using a kit (NextSeq 500/550; Illumina Inc) for 150–base pair paired-end sequencing and has been described previously.^9^ PVs in the following high- or moderate-penetrance genes from panel sequencing were included in the present study: *BRCA1, BRCA2, PALB2, ATM, CHEK2, BARD1, RAD51C, RAD51D,* and *TP53*.

### Statistical Analysis

In the risk estimation model, variables entered the model as either continuous or binary, with the exception of BMI, parity, and height. BMI was modeled as a categorical variable (<18.5, 18.5-24.9, 25-29.9, and ≥30). Parity was modeled with 2 variables, as a linear spline function with a knot at 1 child: first live birth (no or yes) and each additional live birth (continuous variable). Height was modeled as a continuous variable (per 10 cm) and centered at 160 cm.

Mean imputation based on case or control status was performed for the following missing values: family history (n = 1), age at menarche (n = 57), parity (n = 19), height (n = 46), alcohol intake (n = 6), total months of breastfeeding (n = 28), and BMI (n = 74). Sensitivity analyses were conducted in which participants with any missing values for the epidemiologic risk factors were excluded from the analyses.

We calculated a PRS using 46 387 SNVs, based on a previously developed joint and hybrid PRS.^17^ This joint PRS combined a PRS that was trained in women of African ancestry using stepwise logistic regression with a 313-variant PRS that was developed in women of European ancestry.^20^ The hybrid PRS for overall breast cancer combined the PRSs of estrogen receptor–positive and estrogen receptor–negative breast cancer, weighted by subtype proportions. The list of SNVs and corresponding weights used in the PRS calculation are provided in Gao et al.^17^ There were 3054 participants in the present study with information on both genetic and nongenetic factors. Participants whose genetic data were used in the PRS development were excluded from the main analyses (n = 1368) to avoid overfitting the models. This exclusion resulted in a final sample size of 1686 participants.

Missing values in the PVs were recoded to 0 for the main analyses. Sensitivity analyses were performed excluding all participants with missing values in the PVs. In this sensitivity analysis, participants in the PRS development can be included without a contamination issue.

The iCARE (Individualized Coherent Absolute Risk Estimator) R package^21^ was used to estimate the lifetime absolute risk of breast cancer until age 80 years. Breast cancer risk factors were combined using a linear combination of the β coefficients of the questionnaire-based risk factors from the NBCS model,^7^ the β coefficients of the hybrid PRS,^17^ and the meta-analyzed log odds ratios (ORs) for the PVs from the CARRIERS (Cancer Risk Estimates Related to Susceptibility) study^22^ and Breast Cancer Association Consortium.^23^

The following formula was used to account for associations between family history and genetic factors: 
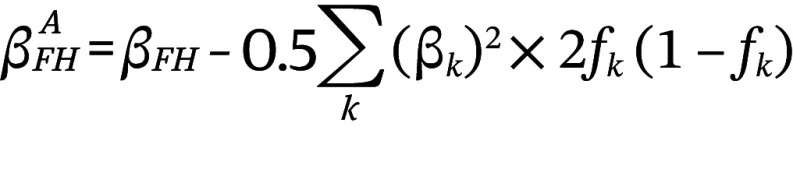
,where *β_k_* is the log OR of the SNVs and PVs unadjusted for family history, *f_k_* is the allele frequency, *β_FH_* is the log OR of family history in the NBCS model, and *β^A^_FH_* is the adjusted log OR of family history. By using this adjustment, the effect size of family history of breast cancer in first-degree relatives is attenuated by a magnitude that is proportional to the degree of heritability explained by the SNVs and high or moderate penetrance PVs in the model.

The model included age-specific breast cancer population incidence rates and mortality rates (excluding breast cancer) obtained from the Ibadan Cancer Registry, which accounts for the competing risk of death from causes other than breast cancer.^24^ Controls from the NBCS were used as the reference dataset for the distribution of risk factors.

Estimated lifetime absolute risk of breast cancer was calculated up to age 80 years for cases and controls. Performance of these lifetime absolute risk estimation models was assessed using area under the receiver operating characteristic curve (AUROC). An AUROC of 1 indicates perfect discriminating performance, and an AUROC of 0.5 indicates no discriminating value. Receiver operating characteristic (ROC) analyses were conducted for models containing epidemiologic risk factors ascertained from NBCS questionnaire only, PRS only, PRS plus PVs, and for the combined model containing all 3 components (epidemiologic risk factors, PRS, and PVs). Because cases and controls had different age distribution and age was associated with multiple risk factors, we also conducted an age-adjusted ROC analysis.

Stata package roccomp was used to calculate unadjusted AUROC,^25^ and Stata package rocreg was used to calculate age-adjusted AUROC.^26^ Analyses were performed from September 2023 to January 2025 using R, version 4.4.1 (R Project for Statistical Computing) and Stata, version 14.2 (StataCorp LLC). Two-sided *P* < .05 indicated statistical significance.

## Results

The main analyses included 1686 women, of whom 996 were cases and 690 were controls, recruited between 1998 and 2018 from Nigeria, Cameroon, and Uganda. The mean (SD) age at study enrollment was 49.5 (12.2) years for cases and 41.5 (13.8) years for controls. As shown in Table 1, most of the participants were recruited from Nigeria (1619 [96.0%]). Compared with controls, cases had a shorter mean (SD) duration of breastfeeding (67.0 [54.3] vs 54.3 [39.4] months); higher proportions of benign breast disease history (3.3% [23] vs 5.1% [51]), breast cancer family history (2.0% [14] vs 3.6% [36]), and alcohol intake (4.8% [33] vs 8.2% [82]); more PVs in genes (eg, in *BRCA1:* 0 vs 27 [2.7%]); and greater mean (SD) PRS (0.182 [0.314] vs 0.268 [0.317]).

**Table 1.** Distribution of Breast Cancer Risk Factors in Cases and Controls

Characteristics	Participants, No. (%)	
	Cases (n = 996)	Controls (n = 690)
Age, y		
Mean (SD)	49.5 (12.2)	41.5 (13.8)
Median (range)	49.0 (20.0 to 89.0)	39.0 (19.0 to 81.0)
Age at menarche, y		
Mean (SD)	15.2 (2.0)	15.3 (2.2)
Median (range)	15.0 (10.0 to 25.0)	15.0 (9.0 to 22.0)
Parity		
Mean (SD)	3.8 (2.1)	3.9 (2.6)
Median (range)	4.0 (0 to 11.0)	4.0 (0 to 14.0)
Total duration of breastfeeding, mo		
Mean (SD)	54.3 (39.4)	67.0 (54.3)
Median (range)	48.0 (0 to 241)	56.7 (0 to 312)
History of benign breast disease		
No	945 (94.9)	667 (96.7)
Yes	51 (5.1)	23 (3.3)
Family history of breast cancer		
No	959 (96.3)	676 (98.0)
Yes	36 (3.6)	14 (2.0)
Missing data	1 (0.1)	0
Height, cm		
Mean (SD)	162 (7.3)	159 (6.6)
Median (range)	162 (126 to 190)	159 (137 to 185)
Weight, kg		
Mean (SD)	69.1 (15.4)	66.9 (15.6)
Median (range)	68.0 (33.0 to 171.0)	65.0 (32.0 to 117.0)
BMI		
<18.5	53 (5.3)	35 (5.1)
18.5-24.9	373 (37.4)	266 (38.6)
25-29.9	280 (28.1)	213 (30.9)
≥30	223 (22.4)	169 (24.5)
Missing data	67 (6.7)	7 (1.0)
Alcohol intake		
No	913 (91.7)	652 (94.5)
Yes	82 (8.2)	33 (4.8)
Missing data	1 (0.1)	5 (0.7)
*BRCA1*		
No	399 (40.1)	406 (58.8)
Yes	27 (2.7)	0
Missing data	570 (57.2)	284 (41.2)
*BRCA2*		
No	408 (41.0)	402 (58.3)
Yes	18 (1.8)	4 (0.6)
Missing data	570 (57.2)	284 (41.2)
*PALB2*		
No	414 (41.6)	406 (58.8)
Yes	7 (0.7)	0
Missing data	575 (57.7)	284 (41.2)
*ATM*		
No	419 (42.1)	406 (58.8)
Yes	2 (0.2)	0
Missing data	575 (57.7)	284 (41.2)
*CHEK2*		
No	421 (42.3)	406 (58.8)
Missing data	575 (57.7)	284 (41.2)
*TP53*		
No	419 (42.1)	406 (58.8)
Yes	2 (0.2)	0
Missing data	575 (57.7)	284 (41.2)
*BARD1*		
No	418 (42.0)	406 (58.8)
Yes	3 (0.3)	0
Missing data	575 (57.7)	284 (41.2)
*RAD51C*		
No	421 (42.3)	406 (58.8)
Missing data	575 (57.7)	284 (41.2)
*RAD51D*		
No	421 (42.3)	406 (58.8)
Missing data	575 (57.7)	284 (41.2)
PRS^a^		
Mean (SD)	0.268 (0.317)	0.182 (0.314)
Median (range)	0.275 (−0.670 to 1.288)	0.193 (−0.793 to 1.148)
Study site		
Cameroon	22 (2.2)	21 (3.0)
Ibadan, Nigeria	845 (84.8)	659 (95.5)
Lagos, Nigeria	114 (11.4)	1 (0.1)
Uganda	15 (1.5)	9 (1.3)

Abbreviations: BMI, body mass index (calculated as weight in kilograms divided by height in meters squared); PRS, polygenic risk score.

^a^ Higher PRS indicates a higher risk of breast cancer.

Age-adjusted AUROC for the absolute risk estimation model was 0.579 (95% CI, 0.549-0.610) with PRS only and increased to 0.609 (95% CI, 0.579-0.638) with PRS plus PVs (Table 2). The age-adjusted AUROC for the epidemiologic risk factors only model was 0.702 (95% CI, 0.676-0.729) and increased to 0.723 (95% CI, 0.698-0.748) with the combined model that incorporated epidemiologic risk factors, PRS, and PVs (Table 2). Sensitivity analyses, in which participants with missing values for the epidemiologic risk factors were excluded from the models, demonstrated similar results, with an age-adjusted AUROC of 0.727 (95% CI, 0.702-0.753) for the combined model (eTable 1 in Supplement 1). Further sensitivity analysis restricted to participants from Nigeria also demonstrated similar results, with an age-adjusted AUROC of 0.739 (95% CI, 0.714-0.763) for the combined model (eTable 2 in Supplement 1). Additionally, sensitivity analysis restricted to participants without missing values in the high- or moderate-penetrance genes (in the entire dataset [n = 1950], including participants who were in the training dataset for PRS) demonstrated an age-adjusted AUROC of 0.682 (95% CI, 0.658-0.706) for the epidemiologic risk factors plus PVs model (eTable 3 in Supplement 1). Since participants who contributed to the training dataset for PRS were included, models with PRS were not evaluated in this sensitivity analysis restricted to participants with no missing values in the high- or moderate-penetrance genes.

**Table 2.** Performance of the Absolute Risk Estimation Models in Participants, With Mean Imputation by Case or Control Status (n = 1686)

Model	AUROC (95% CI)	
	Unadjusted	Age-adjusted
Epidemiologic risk factors only	0.628 (0.601-0.655)	0.702 (0.676-0.729)
PRS only	0.572 (0.544-0.599)	0.579 (0.549-0.610)
PRS plus PVs	0.598 (0.571-0.625)	0.609 (0.579-0.638)
Combined^a^	0.657 (0.631-0.684)	0.723 (0.698-0.748)

Abbreviations: AUROC, area under receiver operating characteristic curve; PRS, polygenic risk score; PV, pathogenic variant.

^a^ Includes epidemiologic risk factors, polygenic risk score, and pathogenic variants.

eFigure in Supplement 1 shows the distribution of estimated lifetime absolute risks for breast cancer in the combined model for controls and cases, with lifetime absolute risk capped at 30%. The mean (SD) estimated lifetime absolute risk was 3.4% (2.7%) for controls and 6.0% (7.5%) for cases. At and beyond the 5% lifetime absolute risk threshold for breast cancer, there was a greater density of cases than controls. Table 3 shows the proportion of participants identified as having a high risk for breast cancer based on different thresholds of lifetime absolute risk. At the 10% or higher threshold, 1.2% of controls (8) and 3.7% of cases (37) were identified as having a high risk in the epidemiologic risk factors only model. Using the PRS plus PV model, 0.6% of controls (4) and 5.0% of cases (50) were identified as having high risk at the 10% or higher threshold. In contrast, in the combined model, 3.3% of controls (23) and 12.0% of cases (120) were identified as having high risk at the 10% or higher threshold.

**Table 3.** Proportion of Participants Identified as Having High Risk of Breast Cancer Based on Threshold of Lifetime Absolute Risk (n = 1686)

Model	Lifetime absolute risk threshold, No. (%)			
	<3%	3%	5%	≥10%
**Epidemiologic risk factors only**				
Controls (n = 690)	431 (62.5)	176 (25.5)	75 (10.9)	8 (1.2)
Cases (n = 996)	449 (45.1)	306 (30.7)	204 (20.5)	37 (3.7)
**PRS only**				
Controls (n = 690)	208 (30.1)	423 (61.3)	59 (8.6)	0
Cases (n = 996)	225 (22.6)	625 (62.8)	146 (14.7)	0
**PRS plus PVs**				
Controls (n = 690)	234 (33.9)	403 (58.4)	49 (7.1)	4 (0.6)
Cases (n = 996)	241 (24.2)	584 (58.6)	121 (12.1)	50 (5.0)
**Combined** ^a^				
Controls (n = 690)	378 (54.8)	189 (27.4)	100 (14.5)	23 (3.3)
Cases (n = 996)	343 (34.4)	284 (28.5)	249 (25.0)	120 (12.0)

Abbreviations: PRS, polygenic risk score; PV, pathogenic variant.

^a^ Includes epidemiologic risk factors, polygenic risk score, and pathogenic variants.

Table 4 shows the distribution of controls and cases at different estimated lifetime absolute risk thresholds by the epidemiologic risk factors only model and demonstrates how this distribution is reclassified by the combined model. At the 3% to 10% threshold in the epidemiologic risk factors only model, 9.6% of controls (24) were reclassified to the lower than 3% threshold, and 6.0% of controls (15) were reclassified to the 10% or higher threshold in the combined model. In contrast, 14.3% of cases (73) were reclassified to the 10% or higher threshold compared with 6.3% of cases (32) being placed within the 3% to 10% threshold in the combined model. When the risk categorization was discordant between the 2 models, the combined model recategorized a greater proportion of cases into the 10% or higher threshold and recategorized a greater proportion of controls into the lower than 3% threshold. The net reclassification index of the combined model was 0.080 or 8.0%. Similarly, the Figure shows that the combined model categorized a higher proportion of participants as having 10% or greater lifetime absolute risk, who would not have been categorized as high risk based on the epidemiologic risk factors only model.

**Table 4.** Reclassification Table of Breast Cancer Risk for Controls and Cases

Epidemiologic risk factors only model	Combined model, No. (%)^a^			
	<3%	3%-10%	≥10%	Row total, No.
**Controls**				
<3%	354 (82.1)	75 (17.4)	2 (0.5)	431
3%-10%	24 (9.6)	212 (84.5)	15 (6.0)	251
≥10%	0	2 (25)	6 (75)	8
Column total, No.	378	289	23	690
**Cases**				
<3%	311 (69.3)	124 (27.6)	14 (3.1)	449
3%-10%	32 (6.3)	405 (79.4)	73 (14.3)	510
≥10%	0	4 (10.8)	33 (89.2)	37
Column total, No.	343	533	120	996

^a^ Includes epidemiologic risk factors, polygenic risk score, and pathogenic variants.

**Figure.**
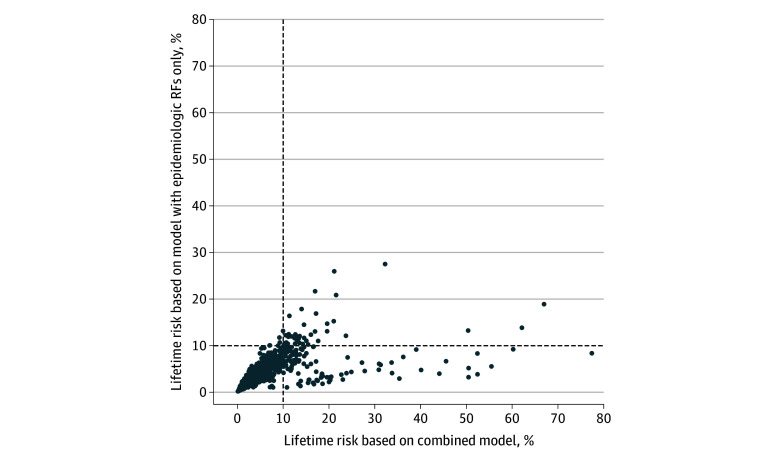
Scatterplot of Estimated Lifetime Absolute Risk of Breast Cancer Based on the Epidemiologic Risk Factors (RFs) Only Model and the Combined Model The combined model includes epidemiologic RFs, polygenic risk score, and pathogenic variants. The dashed lines represent the 10% lifetime absolute risk threshold.

Additional analyses by menopausal status (eTables 4-7 in Supplement 1) showed that the combined model performed similarly for premenopausal and postmenopausal women. We also conducted analyses using the 313 SNV PRS developed in European ancestry populations,^20^ and 248 of the 313 SNVs were available in this analysis. We found that the models with 313 PRS (eTables 8-10 in Supplement 1) performed worse than the models with PRS developed in women of African ancestry. In a sensitivity analysis with PVs restricted to *BRCA1* and *BRCA2* (eTables 11-12 in Supplement 1), we found that the performance of the model with all high or moderate penetrance genes (age-adjusted AUROC = 0.723; 95% CI, 0.698-0.748) was slightly but statistically significantly (*P* = .008) better than the model restricted to *BRCA1* and *BRCA2* (age-adjusted AUROC = 0.719; 95% CI, 0.694-0.744), suggesting that genes other than *BRCA1* and *BRCA2* can reclassify risk for those with PVs in these genes.

## Discussion

By integrating breast cancer risk factors, information on PVs in the high- or moderate-penetrance genes, and a PRS developed with data from the African ancestry training dataset, we built a comprehensive breast cancer risk estimation model for women in Nigeria, Cameroon, and Uganda. This risk estimation model was built using the iCARE package, which allowed us to account for the overlap in the role of family history, PVs, and PRS in breast cancer risk. The NBCS breast cancer risk estimation model, developed for Nigerian women and consisting of epidemiologic risk factors assessed by questionnaire, achieved an age-adjusted AUROC of 0.703 (95% CI, 0.687-0.719) in the Nigerian population, which was superior to the performance of the Gail models and the Black Women’s Health Study model.^7^ In the current study, the combined risk estimation model containing genetic and nongenetic risk factors achieved higher accuracy, with an AUROC of 0.723 (95% CI, 0.698-0.748).

AUROC is just 1 aspect of model performance. Reclassification of women at high risk of breast cancer is more clinically relevant. At multiple risk thresholds, the combined model improved risk stratification and was able to classify a larger proportion of participants as high risk compared with the epidemiologic risk factors only model. In situations where the risk categorization was discordant between the 2 models, the combined model recategorized a greater proportion of cases into the higher risk category and a greater proportion of controls into the lower risk category. The comprehensive risk estimation model we developed integrates information on high- or moderate-penetrance genes, which helps in identifying women at the highest percentiles of breast cancer risk in addition to those with moderately increased risk. Additionally, prior studies have demonstrated the utility of PRS in refining risk estimates for women with PVs in genes with moderate penetrance such as *CHEK2*.^27,28,29^ Compared with clinical management for women carrying PVs in *BRCA1* and *BRCA2*, clinical management for women carrying PVs in other breast cancer genes is less standardized; thus, consideration of this information in conjunction with PRS and epidemiologic risk factors can provide more tailored guidance on how to personalize screening and prevention.

This model’s improved risk stratification has the potential to increase targeted screening and prevention efforts in Nigeria, where most breast cancers are diagnosed at advanced stages (stages III and IV),^2^ by providing a more comprehensive risk assessment tool for genetic counseling, with cascade testing in families with segregating PVs. This capability could enable better identification of individuals and allocation of limited resources to those most likely to benefit from breast imaging and risk-reducing interventions, such as tamoxifen and prophylactic surgery. Estimated frailty-scale heritability of overall breast cancer risk is high in women of African ancestry^19^ compared with women of European ancestry^30^ (0.667 vs 0.515). Therefore, integrating genetic factors is crucial for improving risk stratification in this population. The H3Africa (Human Heredity & Health in Africa) initiative and its H3ABioNet (Pan African Bioinformatics Network) are building capacity for genotyping and genomics data analysis in Africa. Prior studies assessing the implementation of cancer genetic services in Nigeria and Cameroon have demonstrated high willingness by the population to undergo genetic testing, and the majority of people interested in genetic testing are willing to pay for the test.^31,32^

### Strengths and Limitations

Strengths of this study include the use of the original NBCS risk estimation model as a foundation^7,8^ and the integration of a PRS developed for women of African ancestry.^17^ We used population-specific breast cancer incidence rates and competing mortality rates from the Ibadan Cancer Registry to better calibrate absolute risk estimates for the Nigerian population. Additionally, this study can help inform clinical trials assessing personalized screening in diverse populations, such as the WISDOM (Women Informed to Screen Depending on Measures of Risk) trial, by demonstrating the integration of epidemiologic risk factors, PVs, and PRS in a population that has been underrepresented in studies of genetic risk factors but whose ancestral lineage is distributed across the African diaspora.^33^

This study has several limitations that can be addressed in future studies. There was a high proportion of participants for whom high- or moderate-penetrance genes were not genotyped due to limited funds. Breast cancer risk would be underestimated for the participants with an underlying PV but were not genotyped because missing values were imputed to noncarrier status since it is more rare to carry a PV than not. The performance of the risk estimation model is attenuated since the magnitude of underestimation of risk in participants with a PV who were not genotyped is greater for cases, who are more likely than controls to be carriers of a PV. Reliable relative risk estimates for the high or moderate penetrance PVs were not available for Africans. Penetrance estimates from European-ancestry populations may differ from those in African-ancestry populations given that the mean age of the cases in the present study was younger and there was a higher rate of heritability of breast cancer risk.^8^ Additionally, cases and controls were not age-matched, and there may be confounding by age due to birth cohort effect. Older participants are likely to have more lifestyle factors that are protective against breast cancer, such as higher parity and greater duration of breastfeeding, although birth and breastfeeding rates in African countries remain higher than in high-income countries. To understand and account for birth cohort effect, we calculated age-adjusted estimates in the ROC analyses. Additionally, the current model was based on a case-control study; future prospective cohort studies are warranted to calibrate and validate the model.

## Conclusions

Integrating information on genetic factors is a crucial step toward advancing breast cancer risk assessment in sub-Saharan Africa, where breast cancer heritability may be high in the population. We found that the addition of PRS and carrier status of PVs in high- or moderate-penetrance genes to a model containing epidemiologic risk factors improved breast cancer risk stratification in this study of women from Nigeria, Cameroon, and Uganda.

## Notes

Supplement 1. **eFigure.** The Distribution of Lifetime Predicted Absolute Risks for Breast Cancer Cases and Controls in the Combined Model **eTable 1.** Models Without Mean Imputation for the Epidemiological Risk Factors (n = 1,532) **eTable 2.** Models Restricted to Participants From Nigeria (n = 1,551) **eTable 3.** Models With Participants in Whole Dataset, Excluding Participants With Missing Values in the High to Moderate Penetrance Pathogenic Variants (n = 1,950) **eTable 4.** Performance of the Absolute Risk Prediction Models in Premenopausal Study Participants, n = 979 **eTable 5.** Performance of the Absolute Risk Prediction Models in Postmenopausal Study Participants, n = 682 **eTable 6.** Proportion of Premenopausal Participants Identified as High Risk Based on Different Thresholds of Lifetime Risk (n = 979) **eTable 7.** Proportion of Postmenopausal Participants Identified as High Risk Based on Different Thresholds of Lifetime Risk (n = 682) **eTable 8.** Performance of the Absolute Risk Prediction Models With 313 PRS (n = 1,686) **eTable 9.** Proportion of Participants Identified as High Risk Based on Different Thresholds of Lifetime Risk, With 313 PRS (n = 1,686) **eTable 10.** Reclassification Table for Controls and Cases: The Epidemiological Risk Factors Only Model (Rows) and the Combined Risk Factors Model (Columns), With 313 PRS **eTable 11.** Performance of the Absolute Risk Prediction Models in Study Participants, With Information on Pathogenic Variants Restricted to BRCA1 and BRCA2 Versus With Information on Pathogenic Variants of All High/Moderate Penetrance Genes (n = 1,686) **eTable 12.** Reclassification Table for the Combined Model With Information on Pathogenic Variants Restricted to BRCA1 and BRCA2 Versus the Combined Model With Information on Pathogenic Variants of All High/Moderate Penetrance Genes (n = 1,686)

Supplement 2.
Data Sharing Statement
