# Error in the Discussion

**DOI:** 10.1001/jamanetworkopen.2025.19660

**Published:** 2025-06-06

In the Original Investigation titled “Genetic and Nongenetic Risk Factors for Breast Cancer Risk Estimation,”^1^ which was published on April 18, 2025, there was an error in the second paragraph of the Discussion section. *PALB2* was deleted from the following sentence: “Additionally, prior studies have demonstrated the utility of PRS in refining risk estimates for women with PVs in genes with moderate penetrance such as *CHEK2* and *PALB2,*” because some variants in *PALB2* can be considered moderate penetrance while other variants within this gene are more accurately characterized as high penetrance. This article has been corrected.^1^
