# Supplementary material for: Genetic and Nongenetic Risk Factors for Breast Cancer Risk Estimation
Source: JAMA Netw Open. 2025 Apr 18;8(4):e255804. doi: 10.1001/jamanetworkopen.2025.5804 (PMC12008756; doi:10.1001/jamanetworkopen.2025.5804)
Supplement: Supplement 1. — eFigure. The Distribution of Lifetime Predicted Absolute Risks for Breast Cancer Cases and Controls in the Combined Model eTable 1. Models Without Mean Imputation for the Epidemiological Risk Factors (n = 1,532) eTable 2. Models Restricted to Participants From Nigeria (n = 1,551) eTable 3. Models With Participants in Whole Dataset, Excluding Participants With Missing Values in the High to Moderate Penetrance Pathogenic Variants (n = 1,950) eTable 4. Performance of the Absolute Risk Prediction Models in Premenopausal Study Participants, n = 979 eTable 5. Performance of the Absolute Risk Prediction Models in Postmenopausal Study Participants, n = 682 eTable 6. Proportion of Premenopausal Participants Identified as High Risk Based on Different Thresholds of Lifetime Risk (n = 979) eTable 7. Proportion of Postmenopausal Participants Identified as High Risk Based on Different Thresholds of Lifetime Risk (n = 682) eTable 8. Performance of the Absolute Risk Prediction Models With 313 PRS (n = 1,686) eTable 9. Proportion of Participants Identified as High Risk Based on Different Thresholds of Lifetime Risk, With 313 PRS (n = 1,686) eTable 10. Reclassification Table for Controls and Cases: The Epidemiological Risk Factors Only Model (Rows) and the Combined Risk Factors Model (Columns), With 313 PRS eTable 11. Performance of the Absolute Risk Prediction Models in Study Participants, With Information on Pathogenic Variants Restricted to BRCA1 and BRCA2 Versus With Information on Pathogenic Variants of All High/Moderate Penetrance Genes (n = 1,686) eTable 12. Reclassification Table for the Combined Model With Information on Pathogenic Variants Restricted to BRCA1 and BRCA2 Versus the Combined Model With Information on Pathogenic Variants of All High/Moderate Penetrance Genes (n = 1,686) [file jamanetwopen-e255804-s001.pdf]

Supplemental Online Content

Guo W, Li JL, McClellan J, et al. Genetic and nongenetic risk factors and breast cancer among women. *JAMA Netw Open*. Published online April 18, 2025. doi:10.1001/jamanetworkopen.2025.5804

eFigure. The Distribution of Lifetime Predicted Absolute Risks for Breast Cancer Cases and Controls in the Combined Model

eTable 1. Models Without Mean Imputation for the Epidemiological Risk Factors (n = 1,532)

eTable 2. Models Restricted to Participants From Nigeria (n = 1,551)

eTable 3. Models With Participants in Whole Dataset, Excluding Participants With Missing Values in the High to Moderate Penetrance Pathogenic Variants (n=1,950)

eTable 4. Performance of the Absolute Risk Prediction Models in Premenopausal Study Participants, n=979

eTable 5. Performance of the Absolute Risk Prediction Models in Postmenopausal Study Participants, n=682

eTable 6. Proportion of Premenopausal Participants Identified as High Risk Based on Different Thresholds of Lifetime Risk (n=979)

eTable 7. Proportion of Postmenopausal Participants Identified as High Risk Based on Different Thresholds of Lifetime Risk (n= 682)

eTable 8. Performance of the Absolute Risk Prediction Models With 313 PRS (n = 1,686)

eTable 9. Proportion of Participants Identified as High Risk Based on Different Thresholds of Lifetime Risk, With 313 PRS (n=1,686)

eTable 10. Reclassification Table for Controls and Cases: The Epidemiological Risk Factors Only Model (Rows) and the Combined Risk Factors Model (Columns), With 313 PRS

© 2025 Guo W et al. *JAMA Network Open*

eTable 11. Performance of the Absolute Risk Prediction Models in Study Participants, With Information on Pathogenic Variants Restricted to BRCA1 and BRCA2 Versus With Information on Pathogenic Variants of All High/Moderate Penetrance Genes (n = 1,686)

eTable 12. Reclassification Table for the Combined Model With Information on Pathogenic Variants Restricted to BRCA1 and BRCA2 Versus the Combined Model With Information on Pathogenic Variants of All High/Moderate Penetrance Genes (n = 1,686)

This supplemental material has been provided by the authors to give readers additional information about their work.

eFigure. The Distribution of Lifetime Predicted Absolute Risks for Breast Cancer Cases and Controls in the Combined Model

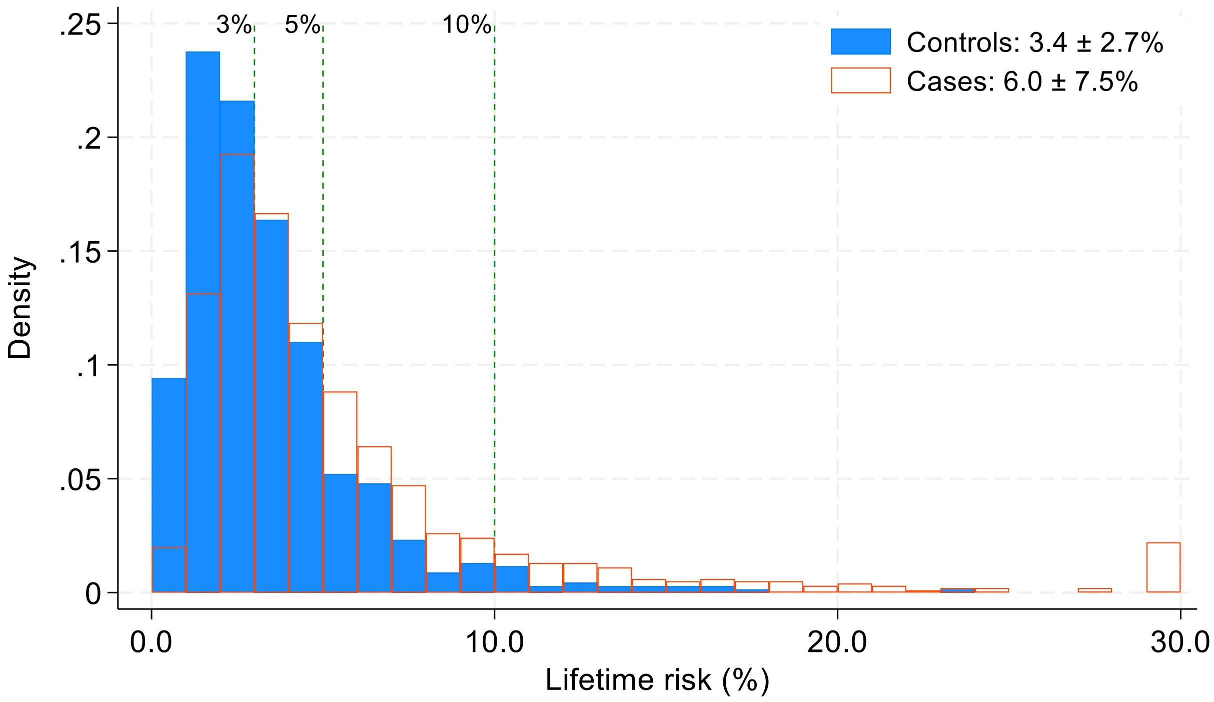

Green dotted lines represent the 3%, 5%, and 10% lifetime absolute risk thresholds for breast cancer

© 2025 Guo W et al. *JAMA Network Open*

eTable 1. Models Without Mean Imputation for the Epidemiological Risk Factors (n = 1,532)

	Unadjusted AUC (95% CI)	Age-adjusted AUC (95% CI)
**Epidemiological RFs only**	0.634 (0.606-0.661)	0.709 (0.682-0.736)
**PRS only**	0.572 (0.544-0.601)	0.579 (0.548-0.610)
**PRS + PVs**	0.599 (0.571-0.627)	0.608 (0.578-0.639)
**Epidemiological RFs + PRS + PVs**	0.662 (0.635-0.689)	0.727 (0.702-0.753)

Abbreviations: PRS, polygenic risk score; RF, risk factor; PVs, pathogenic variants, AUC, area under receiver operating characteristic curve; CI, confidence interval.

© 2025 Guo W et al. *JAMA Network Open*

eTable 2. Models Restricted to Participants From Nigeria (n = 1,551)

	**Unadjusted AUC (95% CI)**	**Age-adjusted AUC (95% CI)**
**Epidemiological RFs only**	0.637 (0.609-0.666)	0.719 (0.693-0.745)
**PRS only**	0.573 (0.544-0.603)	0.584 (0.552-0.616)
**PRS + PVs**	0.600 (0.571-0.629)	0.614 (0.582-0.645)
**Epidemiological RFs + PRS + PVs**	0.666 (0.639-0.694)	0.739 (0.714-0.763)

Abbreviations: PRS, polygenic risk score; RF, risk factor; PVs, pathogenic variants, AUC, area under receiver operating characteristic curve; CI, confidence interval.

© 2025 Guo W et al. *JAMA Network Open*

eTable 3. Models With Participants in Whole Dataset, Excluding Participants With Missing Values in the High to Moderate Penetrance Pathogenic Variants (n=1,950)

	**Unadjusted AUC (95% CI)**	**Age-adjusted AUC (95% CI)**
**Epidemiological RFs only**	0.637 (0.612-0.661)	0.642 (0.617-0.667)
**Epidemiological RFs + PVs**	0.675 (0.651-0.698)	0.682 (0.658-0.706)

Abbreviations: RF, risk factor; PVs, pathogenic variants, AUC, area under receiver operating characteristic curve; CI, confidence interval.

© 2025 Guo W et al. *JAMA Network Open*

eTable 4. Performance of the Absolute Risk Prediction Models in Premenopausal Study Participants, n=979

	**Unadjusted AUC (95% CI)**	**Age-adjusted AUC (95% CI)**
**Epidemiological RFs only**	0.658 (0.624-0.692)	0.721 (0.688-0.754)
**PRS only**	0.553 (0.517-0.589)	0.541 (0.499-0.583)
**PRS + PVs**	0.586 (0.550-0.621)	0.571(0.529-0.614)
**Epidemiological RFs + PRS + PVs (combined model)**	0.676 (0.642-0.709)	0.724 (0.690-0.758)

Abbreviations: PRS, polygenic risk score; RF, risk factor; PVs, pathogenic variants; AUC, area under receiver operating characteristic curve; CI, confidence interval.

© 2025 Guo W et al. *JAMA Network Open*

eTable 5. Performance of the Absolute Risk Prediction Models in Postmenopausal Study Participants, n=682

	**Unadjusted AUC (95% CI)**	**Age-adjusted AUC (95% CI)**
**Epidemiological RFs only**	0.697 (0.651-0.743)	0.698 (0.652-0.744)
**PRS only**	0.627 (0.580-0.675)	0.629(0.582-0.676)
**PRS + PVs**	0.643 (0.597-0.690)	0.642 (0.594-0.690)
**Epidemiological RFs + PRS + PVs (combined model)**	0.735 (0.692-0.778)	0.738 (0.694-0.781)

Abbreviations: PRS, polygenic risk score; RF, risk factor; PVs, pathogenic variants; AUC, area under receiver operating characteristic curve; CI, confidence interval.

© 2025 Guo W et al. *JAMA Network Open*

eTable 6. Proportion of Premenopausal Participants Identified as High Risk Based on Different Thresholds of Lifetime Risk (n=979)

	**Lifetime risk (to age 80)**			
	**<0.03**	**0.03-**	**0.05-**	**≥0.1**
	**N (%)**	**N (%)**	**N (%)**	**N (%)**
**Epidemiological RFs only**				
controls (n=505)	289 (57.23%)	143 (28.32%)	66 (13.07%)	7 (1.39%)
cases (n=474)	163 (34.39%)	163 (34.39%)	119 (25.11%)	29 (6.12%)
**PRS only**				
controls (n=505)	137 (27.13%)	320 (63.37%)	48 (9.50%)	0
cases (n=474)	104 (21.94%)	298 (62.87%)	72 (15.19%)	0
**PRS + PVs**				
controls (n=505)	151 (29.90%)	312 (61.78%)	39 (7.72%)	3 (0.59%)
cases (n=474)	112 (23.63%)	278 (58.65%)	55 (11.60%)	29 (6.12%)
**Epidemiological RFs + PRS + PVs (combined model)**				
controls (n=505)	243 (48.12%)	154 (30.50%)	86 (17.03%)	22 (4.36%)
cases (n=474)	127 (26.79%)	136 (28.69%)	129 (27.22%)	82 (17.30%)

Abbreviations: PRS, polygenic risk score; RF, risk factor; PV, pathogenic variants

© 2025 Guo W et al. *JAMA Network Open*

eTable 7. Proportion of Postmenopausal Participants Identified as High Risk Based on Different Thresholds of Lifetime Risk (n= 682)

	Lifetime risk (to age 80)			
	<0.03	0.03-	0.05-	≥0.1
	N (%)	N (%)	N (%)	N (%)
**Epidemiological RFs only**				
controls (n=176)	139 (77.22%)	33 (18.33%)	7 (3.89%)	1 (0.56%)
cases (n=488)	278 (55.38%)	136 (27.09%)	80 (15.94%)	8 (1.59%)
**PRS only**				
controls (n=176)	68 (37.78%)	101 (56.11%)	11 (6.11%)	0
cases (n=488)	118 (23.51%)	313 (62.35%)	71 (14.14%)	0
**PRS + PVs**				
controls (n=176)	80 (44.44%)	89 (49.44%)	10 (5.56%)	1 (0.56%)
cases (n=488)	126 (25.10%)	293 (58.37%)	64 (12.75%)	19 (3.78%)
**Epidemiological RFs + PRS + PVs (combined model)**				
controls (n=176)	132 (73.33%)	34 (18.89%)	13 (7.22%)	1 (0.56%)
cases (n=488)	212 (42.23%)	141 (28.09%)	114 (22.71%)	35 (6.97%)

Abbreviations: PRS, polygenic risk score; RF, risk factor; PV, pathogenic variants

© 2025 Guo W et al. *JAMA Network Open*

eTable 8. Performance of the Absolute Risk Prediction Models With 313 PRS (n = 1,686)

	**Unadjusted AUC (95% CI)**	**Age-adjusted AUC (95% CI)**
**Epidemiological RFs only**	0.628 (0.601-0.655)	0.702 (0.676-0.729)
**PRS only**	0.550 (0.522-0.577)	0.553 (0.521-0.584)
**PRS + PVs**	0.578 (0.551-0.605)	0.585 (0.554-0.617)
**Epidemiological RFs + PRS + PVs (combined model)**	0.650 (0.624-0.677)	0.720 (0.694-0.745)

Abbreviations: PRS, polygenic risk score; RF, risk factor; PVs, pathogenic variants; AUC, area under receiver operating characteristic curve; CI, confidence interval.

© 2025 Guo W et al. *JAMA Network Open*

eTable 9. Proportion of Participants Identified as High Risk Based on Different Thresholds of Lifetime Risk, With 313 PRS (n=1,686)

	**Lifetime risk (to age 80)**			
	**<0.03**	**0.03-**	**0.05-**	**≥0.1**
	**N (%)**	**N (%)**	**N (%)**	**N (%)**
**Epidemiological RFs only**				
controls (n=690)	431 (62.46%)	176 (25.51%)	75 (10.87%)	8 (1.16%)
cases (n=996)	449 (45.08%)	306 (30.72%)	204 (20.48%)	37 (3.71%)
**PRS only**				
controls (n=690)	306 (44.35%)	384 (55.65%)	0	0
cases (n=996)	368 (36.95%)	627 (62.95%)	1 (0.10%)	0
**PRS + PVs**				
controls (n=690)	351 (50.87%)	335 (48.55%)	4 (0.58%)	0
cases (n=996)	403 (40.46%)	535 (53.71%)	6 (0.60%)	52 (5.22%)
**Epidemiological RFs + PRS + PVs (combined model)**				
controls (n=690)	429 (62.17%)	174 (25.22%)	78 (11.30%)	9 (1.30%)
cases (n=996)	437 (43.88%)	286 (28.71%)	190 (19.08%)	83 (8.33%)

Abbreviations: PRS, polygenic risk score; RF, risk factor; PV, pathogenic variants.

© 2025 Guo W et al. *JAMA Network Open*

eTable 10. Reclassification Table for Controls and Cases: The Epidemiological Risk Factors Only Model (Rows) and the Combined Risk Factors Model (Columns), With 313 PRS

**Epidemiological risk factors only model**	**Combined risk factors model**[a]			
	No. (%)	No. (%)	No. (%)	No.
	Controls			
	<3%	3-10%	≥10%	Column total
<3%	397 (92.11)	32 (7.42)	2 (0.46)	431
3-10%	32 (12.7)	215 (85.7)	4 (1.6)	251
≥10%	0	5 (62.5)	3 (37.5)	8
Row total	429	252	9	690
	Cases			
	<3%	3-10%	≥10%	Column total
<3%	391 (87.1)	45 (10.0)	13 (2.9)	449
3-10%	46 (9.0)	423 (82.9)	41 (8.0)	510
≥10%	0	8 (21.6)	29 (78.4)	37
Row total	437	476	83	996

[a]The combined risk factors model contains epidemiological risk factors, polygenic risk score, and pathogenic variants

eTable 11. Performance of the Absolute Risk Prediction Models in Study Participants, With Information on Pathogenic Variants Restricted to BRCA1 and BRCA2 Versus With Information on Pathogenic Variants of All High/Moderate Penetrance Genes (n = 1,686)

	Unadjusted AUC (95% CI)	Age-adjusted AUC (95% CI)
PRS only	0.572 (0.544-0.599)	0.579 (0.548-0.611)
PRS + PVs of BRCA1/2	0.591 (0.563-0.618)	0.601 (0.572-0.631)
PRS + PVs of all high and moderate penetrance genes	0.598 (0.571-0.625)	0.609 (0.579-0.638)
PRS + PVs of BRCA1/2 + Epidemiological RFs (combined model 1)	0.654 (0.627-0.680)	0.719 (0.694-0.744)
PRS + PVs of all high and moderate penetrance genes + Epidemiological RFs (combined model 2)	0.657 (0.631-0.684)	0.723 (0.698-0.748)

Abbreviations: PRS, polygenic risk score; RF, risk factor; PVs, pathogenic variants; AUC, area under receiver operating characteristic curve; CI, confidence interval.

© 2025 Guo W et al. *JAMA Network Open*

eTable 12. Reclassification Table for the Combined Model With Information on Pathogenic Variants Restricted to BRCA1 and BRCA2 Versus the Combined Model With Information on Pathogenic Variants of All High/Moderate Penetrance Genes (n = 1,686)

**Combined model with BRCA1/2 [a]**	**Combined model with all high/moderate penetrance genes [a]**			
	No. (%)	No. (%)	No. (%)	No.
	Controls [b]			
	<3%	3-10%	≥10%	Column total
<3%	378 (100)	0	0	378
3-10%	0	289 (100)	0	289
≥10%	0	0	23 (100)	23
Row total	378	289	23	690
	Cases			
	<3%	3-10%	≥10%	Column total
<3%	343 (98.56)	**5 (1.44)**	0	348
3-10%	0	528 (98.51)	**8 (1.5)**	536
≥10%	0	0	112 (100)	112
Row total	343	533	120	996

[a] The combined model contains epidemiological risk factors, polygenic risk score, and pathogenic variants
[b] None of controls have pathogenic variants in genes other than BRCA1/2 so no reclassification..

© 2025 Guo W et al. *JAMA Network Open*
