# Supplementary material for: Genetic and Nongenetic Risk Factors for Breast Cancer Risk Estimation
Source: JAMA Netw Open. 2025 Apr 18;8(4):e255804. doi: 10.1001/jamanetworkopen.2025.5804 (PMC12008756; doi:10.1001/jamanetworkopen.2025.5804)
Supplement: Supplement 2. — Data Sharing Statement [file jamanetwopen-e255804-s002.pdf]

Data Sharing Statement

Guo. Genetic and Nongenetic Risk Factors and Breast Cancer Among Women. *JAMA Netw Open*. Published April 18, 2025. doi:10.1001/jamanetworkopen.2025.5804

Data
Data available: Yes
Data types: Deidentified participant data
How to access data: Data will be available via request to corresponding authors Dezheng Huo (dhuo@bsd.uchicago.edu) or Olufunmilayo I. Olopade (folopade@bsd.uchicago.edu)
When available: With publication

Supporting Documents
Document types: None

Additional Information
Who can access the data: Data will be available via request to corresponding authors Dezheng Huo (dhuo@bsd.uchicago.edu) or Olufunmilayo I. Olopade (folopade@bsd.uchicago.edu)
Types of analyses: Data will be available via request to corresponding authors Dezheng Huo (dhuo@bsd.uchicago.edu) or Olufunmilayo I. Olopade (folopade@bsd.uchicago.edu)
Mechanisms of data availability: Data will be available after approval of a proposal and signed data access agreement from corresponding authors Dezheng Huo (dhuo@bsd.uchicago.edu) and Olufunmilayo I. Olopade (folopade@bsd.uchicago.edu)
